# Prognostic Value of the Red Blood Cell Distribution Width-to-Albumin Ratio in Critically Ill Older Patients with Acute Kidney Injury: A Retrospective Database Study

**DOI:** 10.1155/2023/3591243

**Published:** 2023-04-03

**Authors:** Lei Guo, Dezhun Chen, Bihuan Cheng, Yuqiang Gong, Benji Wang

**Affiliations:** Department of Anesthesiology, Critical Care and Pain Medicine, The Second Affiliated Hospital and Yuying Children's Hospital of Wenzhou Medical University, Wenzhou 325000, Zhejiang, China

## Abstract

**Background:**

There is no evidence suggesting that red blood cell distribution width-to-albumin ratio (RA) predicts outcomes in severely ill older individuals with acute kidney injury (AKI). We hypothesized that RA is associated with all-cause mortality in critically ill older patients with AKI.

**Methods:**

We recorded demographics, laboratory tests, comorbidities, vital signs, and other clinical information from the MIMIC-III V1.4 dataset. The primary endpoint was 90-day all-cause mortality, and the secondary endpoints were 30-day mortality, one-year mortality, renal replacement treatment (RRT), duration of stay in the intensive care unit (ICU), sepsis, and septic shock. We generated Cox proportional hazards and logistic regression models to determine RA's prognostic values and subgroup analyses to determine the subgroups' mortality. We conducted a Pearson correlation analysis on RA and C-reactive protein (CRP) in the cohort of patients from the Second Affiliated Hospital of Wenzhou Medical University.

**Results:**

A total of 6,361 patients were extracted from MIMIC-III based on the inclusion and exclusion criteria. RA levels directly and linearly correlated with 90-day all-cause mortality. After controlling for ethnicity, gender, age, and other confounding variables in multivariate analysis, higher RA was significantly associated with an increased risk of 30-day, 90-day, and one-year all-cause mortality as opposed to the reduced levels of RA (tertile 3 vs. tertile 1: hazard ratios (HRs), 95% confidence intervals (CIs): 1.70, 1.43–2.01; 1.90, 1.64–2.19; and 1.95, 1.72–2.20, respectively). These results suggested that elevated levels of RA were linked to an elevated risk of 30-day, 90-day, and one-year all-cause death. There was a similar trend between RA and the use of RRT, length of stay in ICUs, sepsis, and septic shock. The subgroup analysis did not reveal any considerable interplay among strata. When areas under the curve were compared, RA was a weaker predictor than the SAPS II score but a stronger predictor than red blood cell distribution width (RDW) or albumin alone (*P* < 0.001); RA combined with SAPS II has better predictive power than SAPS II alone (*P* < 0.001). The Second Affiliated Hospital of Wenzhou Medical University cohort showed that CRP positively correlated with RA, with a coefficient of 0.2607 (*P* < 0.001).

**Conclusions:**

RA was an independent prognostic predictor in critically ill older patients with AKI, and greater RA was linked to a higher probability of death. The risk of AKI is complicated when RRT occurs; sepsis and septic shock increase with RA levels.

## 1. Introduction

Acute kidney injury (AKI) is diagnosed using serum creatinine and urine output criteria and is linked to morbidity and mortality, particularly in critical diseases [1, 2]. AKI is more likely to occur in critically ill older patients due to age-associated physiological changes, reduced renal reserves, and numerous comorbidities that increase vulnerability to acute renal impairment. Older adults usually use more medications and undergo procedures that might jeopardize renal function [3]. Older illness survivors are frequently unable to regain renal function and rely on long-term dialysis [4], which imposes high financial costs [5]. Given the increasing prevalence of AKI and the dismal outcomes in critical illness, researchers have looked for risk factors for death from AKI [6, 7].

The red blood cell distribution width (RDW) represents the differences in the sizes of circulating red blood cells and is computed in automated complete blood counts [8]. In individuals with cardiovascular diseases [9], multiple myeloma [10], systemic sclerosis [11], and acute ischemic stroke [12], elevated RDW was significantly related to poor outcomes. Our previous study showed that RDW appeared to be an independent prognostic indicator in ill patients with AKI and that elevated RDW was linked to an elevated risk of death in this group [13]; other studies of the MIMIC database have shown that RDW was an independent prognostic factor of long-term outcomes in critically ill patients with AKI [14]. Albumin is an essential protein that regulates osmotic pressure and has anti-inflammatory and antioxidant properties [15, 16]; it has also been linked to AKI [17]. However, whether combining RDW with albumin could predict outcomes in critically sick older patients with AKI is unknown. Therefore, we hypothesized that the red blood cell distribution width-to-albumin ratio (RA) is associated with all-cause mortality in critically ill older patients with AKI.

## 2. Methods

### 2.1. Data Source

We used the methodology of Jia et al. [14, 15]. Multiparameter Intelligent Monitoring in Intensive Care III version 1.4 (MIMIC-III v1.4) is a widely recognized publicly accessible repository containing health information from 40,000 critical care patients between 2001 and 2012 [18]. We completed the Protecting Human Research Participants test and received a certificate (No. 6182750) before applying for database access. Approval of the study was obtained from the Institutional Review Boards of the Massachusetts Institute of Technology and the Beth Israel Deaconess Medical Center with informed consent waivers. In addition, the other part of the data was also obtained from the Second Affiliated Hospital of Wenzhou Medical University from January 2018 to December 2020. Our use of these data was also approved by the Institute of Institutional Research and Ethics of the Second Affiliated Hospital and Yuying Children's Hospital of Wenzhou Medical University (No. LCKY2019-04). Because the data were anonymous, the requirement for informed consent was waived.

### 2.2. Selection Criteria

We limited our search to AKI patients aged 65 years or older. The incidence of AKI was verified using the Kidney Disease: Improving Global Outcomes criteria [19], and additional material included the structured query language for retrieving AKI. Patients had to be included in the ICU for over two days at the time of their initial admission. Exclusion criteria were no RDW and albumin within the first 24 h of admission and over 10% of individual information missing. Subjects in the Second Affiliated Hospital of Wenzhou Medical University cohort also had to meet these criteria.

### 2.3. Data Extraction

The information retried from MIMIC-III included demographics, scoring systems, comorbidities, laboratory tests, vital signs, and other factors recorded. Comorbidities included peripheral vascular disease, sepsis, septic shock, arrhythmia, heart valve disease, diabetes, hypertension, renal failure, hemorrhagic anemia, alcohol abuse, metastatic cancer, solid tumor, and congestive heart failure. Laboratory data included RDW, albumin, prothrombin time (PT), glucose, chloride, potassium, blood urea nitrogen (BUN), sodium, white blood cell count (WBC), platelet, hemoglobin, hematocrit, activated partial thromboplastin time (APTT), international normalized ratio (INR), creatinine, bilirubin, bicarbonate, lactate, and anion gap. RDW was expressed as %, albumin was expressed as g/dL, and RA was calculated as the ratio of RDW to albumin. Other information, including gender, age, systolic blood pressure (SBP), diastolic blood pressure (DBP), renal replacement therapy (RRT), sequential organ failure assessment (SOFA), and simplified acute physiology score II (SAPS II), was also collected.

Our endpoints were 30-day, 90-day, and one-year all-cause mortality beginning from when the patients were admitted to the ICU. The Social Security Death Index records were utilized to acquire vital status survival statistics and calculate mortality rates at different times. The primary endpoint was 90-day all-cause mortality, and the secondary endpoints were 30-day mortality, one-year mortality, RRT, duration of stay in ICUs, sepsis, and septic shock. The data from the Second Affiliated Hospital and Yuying Children's Hospital of Wenzhou Medical University were similarly collected from the medical record system. The collected indicators include clinical parameters, vital signs, laboratory parameters, comorbidities, scoring systems, and mortality (Table S1). Since the number of included cases is not up to the standard, the outcome cannot be directly analyzed, so this portion of the data was placed temporarily into Supplementary Materials.

### 2.4. Statistical Analysis

Continuous variables were expressed as the mean ± SD or interquartile ranges and medians, and categorical variables were expressed as percentages or frequencies. Kruskal–Wallis H tests, one-way analysis of variance, and chi-square tests were performed to determine any significant differences between the cohorts. A generalized additive model was used to determine whether the relationship between RA and 90-day all-cause mortality was linear. Cox proportional hazards models were used to calculate relationships between RA levels and 30-day, 90-day, and one-year all-cause mortality, and the findings were expressed as HRs with 95% CIs. Logistic regression models were used to assess the link among RA and RRT, duration of stay in ICUs, sepsis, and septic shock, showing odds ratios with 95% CIs. As possible confounding factors, variables premised on the biological and epidemiological background were included, and those confounding factors with a change in the effect estimate of more than 10% were utilized to construct an adjusted model [20]. Three multivariate models were created for each endpoint. Stratified linear regression models were utilized to perform subgroup analysis of the relationships between RA and 90-day all-cause mortality. To signify the prognostic efficacy of RA, receiver operating characteristic (ROC) curve analysis was undertaken to compare the area under the ROC curve (AUC). Finally, we conducted Pearson correlation analysis on RA and CRP in the cohort of patients from the Second Affiliated Hospital of Wenzhou Medical University.

All probability estimates were two-sided, with values <0.05 deemed statistically significant. All statistical data were analyzed by using R software (Version 3.6.1).

## 3. Results

### 3.1. Subject Characteristics

A total of 6,361 patients were extracted from MIMIC-III based on the inclusion and exclusion criteria.  Table 1 summarizes the baseline variables of these subjects, split into tertiles according to RA. There were 3,401 men and 2,960 women, and patients with higher RA (RA ≥ 5.68 ml/g) had a higher likelihood of having a history of sepsis, septic shock, peripheral vascular disease, hemorrhagic anemia, coagulopathy, electrolyte disorder, hypothyroidism, congestive heart failure, metastatic cancer, and a solid tumor. Patients in the high-RA cohort (RA ≥ 5.68 ml/g) exhibited high WBC, BUN, chloride, PT, use of RRT, mortality, SOFA score, SAPS II score, lactate, bilirubin, and duration of stay in the ICU. Table S1 in Supplementary Materials shows the baseline variables of patients in the Second Affiliated Hospital of Wenzhou Medical University cohort. Because the number of cases included is not very large, we can only observe the general trend.

### 3.2. RA as a Predictor of Clinical Endpoints

#### 3.2.1. The Linear Association between Levels of RA and 90-Day All-Cause Mortality

**Figure 1:**
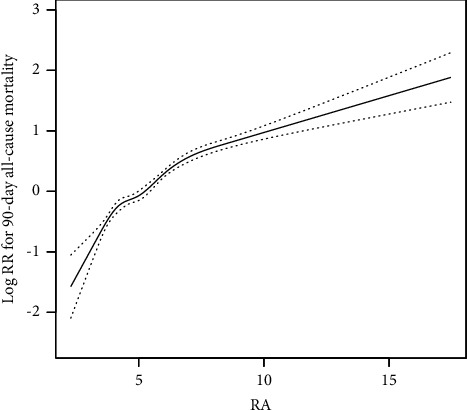
Association between RA and 90-day all-cause mortality.

There was a linear association between levels of RA and 90-day all-cause mortality, with mortality increasing as RA levels increased (Figure 1).

#### 3.2.2. RA Levels Were Related to 30-Day, 90-Day, and One-Year All-Cause Mortality

We used multivariate analysis to determine whether RA levels were related to 30-day, 90-day, and one-year all-cause mortality (Table 2). Upon adjusting for gender, ethnicity, and age in model I, we discovered that elevated RA was linked to a greater risk of death. In model II, upon controlling for ethnicity, age, gender, congestive heart failure, arrhythmia, diabetes, hypertension, renal failure, metastatic cancer, solid tumor, coagulopathy, obesity, electrolyte disorder, hemorrhagic anemia, SpO_2_, temperature, SBP, heart rate, potassium, platelet, chloride, creatinine, bicarbonate, anion gap, hematocrit, BUN, PT, APTT, INR, SAPSII, SOFA, DBP, respiration rate, and WBC, higher RA was significantly associated with an increased risk of 30-day, 90-day, and one-year all-cause mortality as opposed to the reduced levels of RA (tertile 3 vs. tertile 1: HR, 95% CI: 1.70, 1.43–2.01; 1.90, 1.64–2.19; and 1.95, 1.72–2.20, respectively). These results suggested that elevated levels of RA were linked to an elevated risk of 30-day, 90-day, and one-year all-cause death.

#### 3.2.3. The Relationship between RA and Other Endpoints

Various models were developed to evaluate the relationship between RA and the use of RRT, duration of stay in ICUs, sepsis, and septic shock. There were similar trends between RA levels and these partial secondary outcomes (Table 3). The risk of AKI complicated with RRT, sepsis, and septic shock increased with RA levels.

### 3.3. Subgroup Analyses

We undertook subgroup analyses to ascertain whether the connection between RA and the risk of 90-day all-cause mortality was consistent (Table 4). The subgroup analysis revealed no significant interactions, suggesting that the results of this study were stable in these patients.

### 3.4. Mortality Prediction

**Figure 2:**
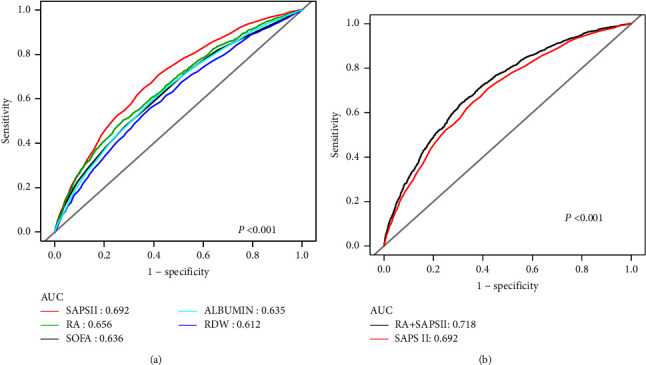
ROC curves for the prediction of 90-day all-cause mortality in critically ill patients with AKI.

According to the ROC curves, the AUCs for RA, RDW, albumin, SAPS II, and SOFA scores were 0.656, 0.612, 0.635, 0.692, and 0.758, respectively (Figure 2(a)). RA was a weaker predictor than the SAPS II score when AUCs were compared, although it was more potent than RDW or albumin alone (*P* < 0.001). SAPS II scores had an AUC of 0.692, contrasted with 0.718 for RA plus SAPS II values (*P* < 0.001). A comparison of the AUCs showed that RA combined with SAPS II has better predictive power than SAPS II alone (Figure 2(b)).

### 3.5. Pearson Correlation Analysis

**Figure 3:**
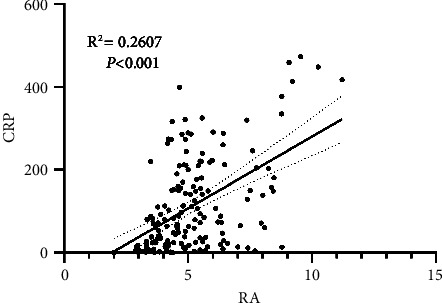
Association between RA and CRP.

We generated CRP and RA scatter plots for patient data from the Second Affiliated Hospital of Wenzhou Medical University. CRP was positively correlated with RA (Figure 3). The correlation coefficient between CRP and RA was 0.2607 (*P* < 0.001).

## 4. Discussion

To the best of our knowledge, there have been no epidemiological studies of the prognostic significance of RA in critically ill older adults with AKI. We found a linear connection between RA levels and 90-day all-cause mortality, with mortality increasing as RA levels increased. Elevated levels of RA were linked to an elevated risk of 30-day, 90-day, and one-year all-cause death in the fully adjusted model. The risk of AKI complicated with RRT, sepsis, and septic shock increased with RA levels. The subgroup analysis indicated that there was no considerable interplay among strata. The ROC curve revealed that RA was lower than the SAPS II value, although it was a stronger predictor than RDW or albumin alone. A comparison of the AUCs showed that RA combined with SAPS II has better predictive power than SAPS II alone. The data obtained from the Second Affiliated Hospital of Wenzhou Medical University cohort showed that RA was positively correlated with CRP in critically ill older individuals with AKI, suggesting that RA may be related to the inflammatory response in these patients.

AKI is a frequent, life-threatening illness with an elevated fatality rate [21]. The etiologies can be classified as prerenal, renal, or postrenal [22]. Prerenal AKI in older adults is caused by volume depletion and reduced effective arterial blood volume, resulting in renal hypoperfusion. Because of the age-associated reduced glomerular filtration rate, reduced renal reserves, and poor autoregulation, vomiting, diarrhea, and excessive diuretic usage induce AKI more commonly and rapidly in older people [23]. In a retrospective investigation of 381 individuals over the age of 80, most (53.5%) had intrinsic AKI, which was primarily caused by shock or prerenal AKI (24.1%) secondary to heart failure and dehydration [24]. Funk et al. found that individuals above 80 years are more likely to develop circulatory AKI due to hypovolemia or shock [25]. Gong et al. found that ischemia was the most significant cause of AKI (53.34%) in patients over 65 [26]. Other intrinsic factors that cause AKI in older people include acute interstitial nephritis, which is frequently caused by hypersensitivity to drugs (especially nonsteroidal anti-inflammatories and antibiotics), glomerulonephritis, and renal vascular disorders [3].

AKI involves a complex physiological process induced by many variables, and its etiology is unknown [27]. A study suggested several theories, one of which is that excessive amounts of inflammatory mediators in the bloodstream significantly contribute to AKI [28]. The inflammatory mediators linked to AKI and its outcome include RDW, albumin concentrations, CRP, TNF-R-II, tumor necrosis factor receptor I, interleukin- (IL-) 6, IL-10, platelets, lymphocytes, and neutrophils [29, 30]. Impairment of the autoregulation of renal blood flow is another potential cause of AKI [31]. Reduced renal blood flow exhausts intracellular adenosine triphosphate, compromises the cytoskeleton's integrity, triggers inflammatory pathways, produces free radicals, and disrupts intracellular calcium homeostasis [32, 33]. These lesions cause hypoxic damage to tubular cells, resulting in casts that block renal tubules.

Our previous study illustrated that RDW was among these biomarkers for the outcome of AKI [13]. RDW is a readily accessible biomarker to predict the development of various illnesses and organ dysfunctions [34, 35]. In several observational studies, investigators have shown a link between elevated RDW and alterations in inflammatory biomarkers [36, 37]. These findings suggest that the systemic inflammatory response is instrumental in explaining the possible connection between RDW and mortality in severely ill AKI patients. Moreover, according to several research reports, hypoalbuminemia has also been linked to the progression of AKI and poor outcomes in patients with severe illnesses [38, 39]. Albumin protects the kidneys from toxins and keeps glial pressure at optimal levels for adequate renal perfusion [40]. The present study results suggest that RA is a stronger independent predictor of all-cause mortality in critically ill older patients with AKI than albumin or RDW alone, and we have grounds to assume that RA is clinically significant.

There were some limitations in our research. First, biases were unavoidable because this was a retrospective study based on only one center. Second, we computed RA only after the patient was admitted to the ICU, and only one measure of RA might alter the results' accuracy. Third, despite our best efforts to minimize bias using a multivariate model, countless additional known and unknown variables exist. Finally, retrospective databases entail numerous flaws; hence, multicenter prospective studies must validate our findings.

## 5. Conclusions

RA was an independent prognostic predictor in critically ill older patients with AKI, and elevated RA was linked to a greater risk of death among these patients. The risk of AKI complicated with RRT, sepsis, and septic shock increased with RA levels. Extensive prospective multicenter investigations are needed to validate these results.

## Figures and Tables

**Table 1:** Baseline characteristics of the study population.

Characteristics	RA level (ml/g)			*P* value
	<4.35 (*n* = 2117)	≥4.35, <5.68 (*n* = 2119)	≥5.68 (*n* = 2125)	
Clinical parameters				
Age, years	78.1 ± 7.9	78.2 ± 7.6	77.5 ± 7.6	0.005
Gender, *n* (%)				0.023
Female	941 (44.4)	1031 (48.7)	988 (46.5)	
Male	1176 (55.6)	1088 (51.3)	1137 (53.5)	
Ethnicity, *n* (%)				0.004
White	1585 (74.9)	1550 (73.1)	1507 (70.9)	
Black	164 (7.7)	217 (10.2)	205 (9.6)	
Other	368 (17.4)	352 (16.6)	413 (19.4)	
Vital signs				
SBP (mmHg)	121.9 ± 18.0	116.8 ± 17.4	112.2 ± 15.4	<0.001
DBP (mmHg)	59.1 ± 10.2	56.5 ± 9.4	54.5 ± 9.3	<0.001
MAP (mmHg)	78.0 ± 11.0	74.5 ± 10.3	71.9 ± 9.8	<0.001
Heart rate (beats/minute)	81.0 ± 15.1	84.4 ± 16.6	87.7 ± 16.0	<0.001
Respiratory rate (times/minute)	19.2 ± 3.8	19.8 ± 4.1	19.9 ± 4.4	<0.001
Temperature (°C)	36.8 ± 0.6	36.7 ± 0.7	36.6 ± 0.7	<0.001
SpO_2_ (%)	97.1 ± 2.3	97.0 ± 2.3	97.0 ± 3.5	0.718
Comorbidities				
Congestive heart failure, *n* (%)	548 (25.9)	828 (39.1)	1181 (55.6)	<0.001
Sepsis, *n* (%)	192 (9.1)	412 (19.4)	653 (30.7)	<0.001
Septic shock, *n* (%)	117 (5.5)	282 (13.3)	430 (20.2)	<0.001
Arrhythmia, *n* (%)	890 (42.0)	990 (46.7)	955 (44.9)	0.008
Heart valve disease, *n* (%)	398 (18.8)	399 (18.8)	301 (14.2)	<0.001
Peripheral vascular disease, *n* (%)	224 (10.6)	285 (13.4)	308 (14.5)	<0.001
Diabetes, *n* (%)	544 (25.7)	587 (27.7)	503 (23.7)	0.011
Hypertension, *n* (%)	1495 (70.6)	1386 (65.4)	1212 (57.0)	0.174
Renal failure, *n* (%)	443 (20.9)	678 (32.0)	671 (31.6)	<0.001
Hemorrhagic anemia, *n* (%)	42 (2.0)	62 (2.9)	89 (4.2)	<0.001
Alcohol abuse, *n* (%)	83 (3.9)	59 (2.8)	72 (3.4)	0.122
Coagulopathy, *n* (%)	219 (10.3)	339 (16.0)	533 (25.1)	<0.001
Electrolyte disorder, *n* (%)	792 (37.4)	977 (46.1)	1077 (50.7)	<0.001
Nervous system diseases, *n* (%)	339 (16.0)	290 (13.7)	207 (9.7)	<0.001
Hypothyroidism, *n* (%)	237 (11.2)	284 (13.4)	316 (14.9)	0.002
Metastatic cancer, *n* (%)	37 (1.7)	117 (5.5)	208 (9.8)	<0.001
Solid tumor, *n* (%)	73 (3.4)	88 (4.2)	127 (6.0)	<0.001
Obesity, *n* (%)	71 (3.4)	86 (4.1)	82 (3.9)	0.462
Laboratory parameters				
RA level (ml/g)	3.8 ± 0.4	5.0 ± 0.4	7.2 ± 1.6	<0.001
RDW (%)	14.1 ± 1.1	15.5 ± 1.6	17.2 ± 2.4	<0.001
Albumin (g/dL)	3.8 ± 0.4	3.1 ± 0.4	2.5 ± 0.5	<0.001
White blood cell count (10^9^/L)	12.4 ± 7.6	13.1 ± 11.0	15.4 ± 17.6	<0.001
Platelet (10^9^/L)	252.1 ± 104.9	254.7 ± 135.0	253.8 ± 154.0	0.818
Hemoglobin (g/dL)	12.6 ± 1.9	11.4 ± 1.8	10.9 ± 1.8	<0.001
Hematocrit (%)	37.5 ± 5.5	34.5 ± 5.4	33.2 ± 5.2	<0.001
Creatinine (mg/dL)	1.8 ± 1.6	2.3 ± 1.9	2.2 ± 1.7	<0.001
BUN (mg/dL)	35.8 ± 24.9	44.8 ± 28.2	46.6 ± 29.9	<0.001
Sodium (mmol/L)	140.7 ± 5.3	140.7 ± 5.6	140.8 ± 5.8	0.850
Potassium (mmol/L)	4.8 ± 1.0	4.9 ± 1.0	4.8 ± 1.0	0.146
Chloride (mmol/L)	106.6 ± 6.8	107.2 ± 7.2	108.8 ± 7.5	<0.001
Glucose (mg/dL)	198.9 ± 111.6	199.3 ± 132.0	185.7 ± 103.5	<0.001
Bilirubin (mg/dL)	1.0 ± 1.5	1.2 ± 2.0	2.2 ± 4.7	<0.001
Bicarbonate (mmol/L)	25.5 ± 4.4	24.6 ± 5.0	23.7 ± 5.1	<0.001
PT (second)	17.6 ± 11.9	19.7 ± 13.9	20.3 ± 14.2	<0.001
INR	1.8 ± 1.6	2.1 ± 2.5	2.1 ± 2.1	<0.001
APTT (second)	49.2 ± 36.7	47.8 ± 33.3	50.7 ± 32.8	<0.001
Lactate (mmol/L)	3.1 ± 2.3	3.3 ± 2.9	3.7 ± 3.2	<0.001
Anion gap (mmol/L)	17.6 ± 4.6	17.8 ± 4.8	17.3 ± 5.0	<0.001
Scoring systems				
SAPSII	40.9 ± 11.3	45.6 ± 12.9	50.3 ± 14.5	<0.001
SOFA	4.4 ± 2.7	5.6 ± 3.1	6.8 ± 3.6	<0.001
Mortality				
30-day, *n* (%)	282 (13.3)	400 (18.9)	668 (31.4)	<0.001
90-day, *n* (%)	385 (18.2)	566 (26.7)	923 (43.4)	<0.001
1-year, *n* (%)	548 (25.9)	828 (39.1)	1181 (55.6)	<0.001
Length of stay in ICU, day	4.9 ± 6.3	5.6 ± 6.7	6.9 ± 8.8	<0.001
Renal replacement therapy, *n* (%)	106 (5.0)	234 (11.0)	336 (15.8)	<0.001

RA: the ratio of red cell volume distribution width to albumin; SBP: systolic blood pressure; DBP: diastolic blood pressure; MAP: mean arterial pressure; RDW: red cell volume distribution width; BUN: blood urea nitrogen; PT: prothrombin time; INR: international normalized ratio; APTT: activated partial thromboplastin time; SAPSII: simplified acute physiology score II; SOFA: sequential organ failure assessment; ICU: intensive care unit.

**Table 2:** HRs (95% CIs) for all-cause mortality across groups of RA level.

RA level (ml/g)	Nonadjusted		Model I		Model II	
	HR (95% CIs)	*P* value	HR (95% CIs)	*P* value	HR (95% CIs)	*P* value
Primary outcomes						
90-day all-cause mortality						
Continuous variable	1.25 (1.22, 1.27)	<0.0001	1.26 (1.24, 1.29)	<0.0001	1.17 (1.14, 1.20)	<0.0001
Tertiles						
<4.35	1.0 (ref)		1.0 (ref)		1.0 (ref)	
≥4.35, <5.68	1.54 (1.35, 1.75)	<0.0001	1.56 (1.37, 1.78)	<0.0001	1.22 (1.06, 1.41)	0.0049
≥5.68	2.80 (2.49, 3.16)	<0.0001	2.88 (2.56, 3.24)	<0.0001	1.90 (1.64, 2.19)	<0.0001
*P* trend	<0.0001		<0.0001		<0.0001	
Secondary outcomes						
30-day all-cause mortality						
Continuous variable	1.25 (1.22, 1.28)	<0.0001	1.26 (1.23, 1.29)	<0.0001	1.15 (1.12, 1.19)	<0.0001
Tertiles						
<4.35	1.0 (ref)		1.0 (ref)		1.0 (ref)	
≥4.35, <5.68	1.46 (1.25, 1.70)	<0.0001	1.48 (1.27, 1.72)	<0.0001	1.15 (0.97, 1.35)	0.1019
≥5.68	2.62 (2.28, 3.01)	<0.0001	2.67 (2.33, 3.07)	<0.0001	1.70 (1.43, 2.01)	<0.0001
*P* trend	<0.0001		<0.0001		<0.0001	
1-year all-cause mortality						
Continuous variable	1.24 (1.22, 1.26)	<0.0001	1.25 (1.23, 1.27)	<0.0001	1.17 (1.15, 1.20)	<0.0001
Tertiles						
<4.35	1.0 (ref)		1.0 (ref)		1.0 (ref)	
≥4.35, <5.68	1.63 (1.46, 1.81)	<0.0001	1.65 (1.48, 1.84)	<0.0001	1.32 (1.18, 1.49)	<0.0001
≥5.68	2.72 (2.46, 3.01)	<0.0001	2.80 (2.53, 3.10)	<0.0001	1.95 (1.72, 2.20)	<0.0001
*P* trend	<0.0001		<0.0001		<0.0001	

HR: hazard ratio; CI: confidence interval. Models were derived from Cox proportional hazards regression models. Nonadjusted model adjusting for none. Adjusted model I adjusting for age, ethnicity, and gender. Adjusted model II adjusting for congestive heart failure, arrhythmia, age, ethnicity, gender, diabetes, hypertension, renal failure, metastatic cancer, solid tumor, coagulopathy, obesity, electrolyte disorder, hemorrhagic anemia, anion gap, bicarbonate, creatinine, chloride, hematocrit, platelet, Potassium, APTT, INR, PT, BUN, SAPSII, SOFA, heart rate, systolic blood pressure, diastolic blood pressure, respiration rate, temperature, SpO_2_, and white blood cell count.

**Table 3:** ORs (95% CIs) for partial secondary outcomes across RA levels.

	Nonadjusted		Model I		Model II	
	OR (95% CIs)	*P* value	OR (95% CIs)	*P* value	OR (95% CIs)	*P* value
Renal replacement therapy	1.24 (1.19, 1.29)	<0.0001	1.24 (1.19, 1.29)	<0.0001	1.27 (1.18, 1.35)	<0.0001
Length of stay in ICU	0.50 (0.40, 0.61)	<0.0001	0.49 (0.39, 0.60)	<0.0001	0.19 (0.07, 0.32)	0.0027
Sepsis	1.34 (1.29, 1.38)	<0.0001	1.35 (1.30, 1.40)	<0.0001	1.12 (1.07, 1.18)	<0.0001
Septic shock	1.31 (1.26, 1.36)	<0.0001	1.32 (1.27, 1.37)	<0.0001	1.08 (1.02, 1.13)	0.0043

OR: odds ratio; CI: confidence interval. Models were derived from logistic regression models. Nonadjusted model adjusting for none. Adjusted model I adjusting for age, ethnicity, and gender. Adjusted model II adjusting for congestive heart failure, arrhythmia, age, ethnicity, gender, diabetes, hypertension, renal failure, metastatic cancer, solid tumor, coagulopathy, obesity, electrolyte disorder, hemorrhagic anemia, anion gap, bicarbonate, creatinine, chloride, hematocrit, platelet, potassium, APTT, INR, PT, BUN, SAPSII, SOFA, heart rate, systolic blood pressure, diastolic blood pressure, respiration rate, temperature, SpO_2_, and white blood cell count.

**Table 4:** Subgroup analysis of the associations between 90-day all-cause mortality and the RA level.

	No. of patients	HR (95% CI)	*P* value
Age (year)			
<77.7	3180	1.25 (1.21, 1.28)	<0.0001
≥77.7	3181	1.27 (1.23, 1.30)	<0.0001
Gender			
Female	2960	1.24 (1.21, 1.28)	<0.0001
Male	3401	1.26 (1.22, 1.30)	<0.0001
Ethnicity			
White	4642	1.25 (1.22, 1.29)	<0.0001
Black	586	1.23 (1.16, 1.30)	<0.0001
Other	1133	1.25 (1.20, 1.30)	<0.0001
Sepsis			
No	5104	1.23 (1.20, 1.26)	<0.0001
Yes	1257	1.23 (1.18, 1.28)	<0.0001
Heart rate (beats/minute)			
<83	3173	1.29 (1.25, 1.33)	<0.0001
≥83	3174	1.21 (1.18, 1.24)	<0.0001
SBP (mmHg)			
<114	3171	1.22 (1.20, 1.26)	<0.0001
≥114	3171	1.24 (1.19, 1.29)	<0.0001
DBP (mmHg)			
<56	3170	1.24 (1.21, 1.27)	<0.0001
≥56	3172	1.25 (1.21, 1.29)	<0.0001
MAP (mmHg)			
<73	3173	1.25 (1.21, 1.28)	<0.0001
≥73	3174	1.23 (1.19, 1.27)	<0.0001
Respiratory rate (times/minute)			
<19	3170	1.26 (1.22, 1.30)	<0.0001
≥19	3170	1.23 (1.20, 1.27)	<0.0001
SpO_2_ (%)			
<97.5	3168	1.27 (1.24, 1.31)	<0.0001
≥97.5	3174	1.23 (1.19, 1.26)	<0.0001
Temperature (°C)			
<36.7	3139	1.25 (1.22, 1.28)	<0.0001
≥36.7	3146	1.24 (1.20, 1.28)	<0.0001
Bicarbonate (mg/dL)			
<24	2654	1.21 (1.18, 1.25)	<0.0001
≥24	3702	1.27 (1.24, 1.30)	<0.0001
Bilirubin (mg/dL)			
<0.6	2245	1.26 (1.22, 1.30)	<0.0001
≥0.6	3204	1.26 (1.22, 1.29)	<0.0001
Glucose (mg/dL)			
<166	3169	1.26 (1.23, 1.30)	<0.0001
≥166	3186	1.24 (1.21, 1.28)	<0.0001
WBC (10^9^/L)			
<11.5	3167	1.27 (1.23, 1.30)	<0.0001
≥11.5	3194	1.22 (1.19, 1.26)	<0.0001
Hematocrit (%)			
<34.4	3143	1.24 (1.21, 1.28)	<0.0001
≥34.4	3215	1.27 (1.23, 1.31)	<0.0001
Hemoglobin (g/dL)			
<11.4	3096	1.23 (1.20, 1.26)	<0.0001
≥11.4	3262	1.27 (1.23, 1.31)	<0.0001
Platelet (10^9^/L)			
<216	3177	1.25 (1.22, 1.29)	<0.0001
≥216	3180	1.24 (1.21, 1.28)	<0.0001
Sodium (mg/dL)			
<141	3147	1.24 (1.20, 1.27)	<0.0001
≥141	3209	1.26 (1.22, 1.29)	<0.0001
Potassium (mmol/L)			
<4.6	2960	1.26 (1.22, 1.29)	<0.0001
≥4.6	3398	1.24 (1.21, 1.28)	<0.0001
Chloride (mg/dL)			
<107	2837	1.26 (1.22, 1.30)	<0.0001
≥107	3519	1.25 (1.22, 1.28)	<0.0001
PT (second)			
<15.3	3032	1.23 (1.19, 1.28)	<0.0001
≥15.3	3083	1.24 (1.21, 1.27)	<0.0001
INR			
<1.4	2722	1.25 (1.20, 1.30)	<0.0001
≥1.4	3392	1.23 (1.20, 1.26)	<0.0001
APTT (second)			
<35.2	3046	1.29 (1.24, 1.34)	<0.0001
≥35.2	3055	1.22 (1.20, 1.25)	<0.0001
Creatinine (mg/dL)			
<1.5	2940	1.27 (1.23, 1.32)	<0.0001
≥1.5	3418	1.22 (1.19, 1.25)	<0.0001
BUN (mg/dL)			
<34	3078	1.25 (1.21, 1.29)	<0.0001
≥34	3280	1.23 (1.20, 1.26)	<0.0001
Anion gap (mg/dL)			
<17	3011	1.25 (1.21, 1.29)	<0.0001
≥17	3321	1.27 (1.24, 1.31)	<0.0001
Albumin (g/dL)			
<3.1	2886	1.20 (1.17, 1.23)	<0.0001
≥3.1	3475	1.40 (1.29, 1.51)	<0.0001
RDW (%)			
<15.1	3052	1.31 (1.25, 1.37)	<0.0001
≥15.1	3309	1.21 (1.18, 1.24)	<0.0001
Lactate (mmol/L)			
<2.4	2290	1.25 (1.20, 1.29)	<0.0001
≥2.4	2406	1.20 (1.16, 1.23)	<0.0001
Sepsis			
No	5104	1.23 (1.20, 1.26)	<0.0001
Yes	1257	1.23 (1.18, 1.28)	<0.0001
Septic shock			
No	5532	1.24 (1.21, 1.27)	<0.0001
Yes	829	1.21 (1.16, 1.27)	<0.0001
Congestive heart failure			
No	3566	1.22 (1.19, 1.26)	<0.0001
Yes	2795	1.30 (1.26, 1.34)	<0.0001
Arrhythmia			
No	3526	1.25 (1.21, 1.28)	<0.0001
Yes	2835	1.26 (1.22, 1.30)	<0.0001
Heart valve disease			
No	5263	1.25 (1.23, 1.28)	<0.0001
Yes	1098	1.23 (1.16, 1.30)	<0.0001
Peripheral vascular disease			
No	5544	1.26 (1.23, 1.29)	<0.0001
Yes	817	1.18 (1.12, 1.25)	<0.0001
Hypertension			
No	2268	1.23 (1.19, 1.27)	<0.0001
Yes	4093	1.25 (1.22, 1.29)	<0.0001
Nervous system diseases			
No	5525	1.26 (1.23, 1.28)	<0.0001
Yes	836	1.18 (1.10, 1.26)	<0.0001
Diabetes			
No	4727	1.24 (1.21, 1.26)	<0.0001
Yes	1634	1.31 (1.25, 1.38)	<0.0001
Hypothyroidism			
No	5524	1.25 (1.22, 1.27)	<0.0001
Yes	837	1.25 (1.18, 1.32)	<0.0001
Renal failure			
No	4569	1.27 (1.24, 1.30)	<0.0001
Yes	1792	1.20 (1.15, 1.24)	<0.0001
Metastatic cancer			
No	5999	1.25 (1.23, 1.28)	<0.0001
Yes	362	1.08 (1.00, 1.16)	0.0465
Solid tumor			
No	6073	1.25 (1.22, 1.27)	<0.0001
Yes	288	1.30 (1.20, 1.41)	<0.0001
Coagulopathy	2952	1.27 (1.20, 1.35)	
No	5270	1.26 (1.23, 1.29)	<0.0001
Yes	1091	1.20 (1.15, 1.25)	<0.0001
Obesity			
No	6122	1.25 (1.22, 1.27)	<0.0001
Yes	239	1.36 (1.18, 1.57)	<0.0001
Electrolyte disorder			
No	3515	1.28 (1.24, 1.31)	<0.0001
Yes	2846	1.22 (1.18, 1.25)	<0.0001
Hemorrhagic anemia			
No	6168	1.25 (1.22, 1.27)	<0.0001
Yes	193	1.27 (1.12, 1.46)	0.0004
Alcohol abuse			
No	6147	1.25 (1.22, 1.27)	<0.0001
Yes	214	1.28 (1.11, 1.46)	0.0004
SAPSII			
<44	3158	1.27 (1.22, 1.32)	<0.0001
≥44	3203	1.17 (1.14, 1.20)	<0.0001
SOFA			
<5	2662	1.29 (1.23, 1.36)	<0.0001
≥5	3699	1.20 (1.17, 1.23)	<0.0001
AKI stage			
I	1514	1.30 (1.25, 1.35)	<0.0001
II	944	1.18 (1.10, 1.25)	<0.0001
III	3903	1.24 (1.21, 1.27)	<0.0001

SBP: systolic blood pressure; DBP: diastolic blood pressure; MAP: mean arterial pressure; RA: the ratio of red cell volume distribution width to albumin; RDW: red cell volume distribution width; CRP: C-reactive protein; PT: prothrombin time; INR: international normalized ratio; APTT: activated partial thromboplastin time; BUN: blood urea nitrogen; COPD: chronic obstructive pulmonary disease; ARDS: acute respiratory distress syndrome; AKI: acute kidney injury; SAPSII: simplified acute physiology score II; SOFA: sequential organ failure assessment; ICU: intensive care unit.

## Data Availability

The clinical data for this study were provided by MIMIC-III v.1.4. Researchers must complete the National Institutes of Health's online course Protecting Human Research Participants to apply for permission to access the database. The other part of the data was also obtained from the Second Affiliated Hospital of Wenzhou Medical University; due to the small number of participants and specific nature of the service, the data would not be shared for the time being.

## Supplementary Materials

Supplementary Materials The baseline variables of the study population in the Second Affiliated Hospital of Wenzhou Medical University cohort (Table S1).
