# Supplementary material for: Prognostic Value of the Red Blood Cell Distribution Width-to-Albumin Ratio in Critically Ill Older Patients with Acute Kidney Injury: A Retrospective Database Study
Source: Emerg Med Int. 2023 Apr 3;2023:3591243. doi: 10.1155/2023/3591243 (PMC10085652; doi:10.1155/2023/3591243)
Supplement: Supplementary Materials — The baseline variables of the study population in the Second Affiliated Hospital of Wenzhou Medical University cohort (Table S1). [file 3591243.f1.docx]

**Table S1** Baseline characteristics of the study population from the second affiliated hospital of Wenzhou Medical University

| Characteristics | RA level, ml/g | | |
| --- | --- | --- | --- |
|  | <4.42(n=38) | ≥4.42, <5.49 (n=37) | ≥5.49 (n=38) |
| **Clinical parameters** |  |  |  |
| Age, years | 76.05 ± 7.69 | 78.21 ± 7.16 | 79.46 ± 7.75 |
| Gender, n (%) |  |  |  |
| Female | 11 (28.95) | 15 (40.54) | 9 (23.68) |
| Male | 27 (71.05) | 22 (59.46) | 29 (76.32) |
| **Vital signs** |  |  |  |
| Temperature, ℃ | 37.09 ± 1.22 | 37.28 ± 1.05 | 36.52 ± 1.56 |
| Heart rate, beats/minute | 100.39 ± 24.25 | 104.00 ± 23.87 | 104.68 ± 21.82 |
| Respiratory rate, times/minute | 21.11 ± 5.48 | 24.30 ± 16.66 | 21.32 ± 4.78 |
| SpO2, ％ | 96.37 ± 4.39 | 97.51 ± 4.04 | 96.43 ± 3.63 |
| SBP, mmHg | 127.34 ± 35.31 | 117.43 ± 32.05 | 122.89 ± 29.80 |
| DBP, mmHg | 73.13 ± 16.74 | 71.14 ± 17.31 | 66.84 ± 16.68 |
| MAP, mmHg | 91.20 ± 21.06 | 86.57 ± 19.14 | 83.27 ± 23.44 |
| **Laboratory parameters** |  |  |  |
| RA level, ml/g | 3.67 ± 0.51 | 4.88 ± 0.29 | 6.95 ± 1.29 |
| RDW, % | 13.44 ± 0.95 | 14.46 ± 1.31 | 16.42 ± 3.35 |
| Albumin, g/dL | 37.35 ± 6.14 | 29.71 ± 3.17 | 23.97 ± 4.29 |
| PH | 7.35 ± 0.08 | 7.34 ± 0.10 | 7.32 ± 0.11 |
| Bicarbonate, mmol/L | 17.71 ± 4.59 | 18.79 ± 5.28 | 16.56 ± 5.69 |
| Anion gap, mmol/L | 20.06 ± 5.67 | 18.35 ± 5.21 | 18.30 ± 5.73 |
| Lactate, mmol/L | 3.46 ± 3.18 | 3.77 ± 3.70 | 4.07 ± 3.16 |
| Glucose, mmol/L | 9.75 ± 4.62 | 10.96 ± 5.36 | 11.16 ± 5.02 |
| CRP, mg/L | 108.56 ± 95.76 | 119.15 ± 93.27 | 127.32 ± 103.44 |
| White blood cell count, 10^9^/L | 13.51 ± 5.61 | 13.86 ± 7.79 | 18.75 ± 8.10 |
| Hemoglobin, g/dL | 128.05 ± 23.41 | 104.16 ± 23.51 | 92.63 ± 27.94 |
| Hematocrit, % | 0.38 ± 0.07 | 0.31 ± 0.08 | 0.28 ± 0.09 |
| Platelet, 10^9^/L | 203.00 ± 77.14 | 174.79 ± 119.30 | 150.57 ± 135.75 |
| PT, second | 15.41 ± 3.36 | 18.16 ± 5.83 | 20.69 ± 8.90 |
| INR | 1.26 ± 0.39 | 1.57 ± 0.67 | 1.84 ± 1.15 |
| APTT, second | 39.56 ± 8.65 | 48.72 ± 16.87 | 56.22 ± 24.55 |
| D – dimer, μg/mL | 7.54 ± 7.60 | 13.97 ± 23.88 | 13.69 ± 33.07 |
| cTnI, ng/mL | 1.76 ± 3.84 | 1.57 ± 4.53 | 1.41 ± 4.89 |
| Nt-proBNP, pg/L | 6343.31 ± 5306.26 | 9396.62 ± 8702.38 | 9503.09 ± 8242.67 |
| Bilirubin, mmol/L | 19.07 ± 13.68 | 29.70 ± 49.23 | 28.15 ± 47.79 |
| BUN, mmol/L | 11.79 ± 6.16 | 17.42 ± 8.29 | 16.29 ± 12.63 |
| Creatinine, mmol/L | 175.86 ± 128.51 | 219.68 ± 120.01 | 240.95 ± 150.05 |
| Sodium, mmol/L | 141.55 ± 9.99 | 141.85 ± 10.04 | 140.38 ± 9.55 |
| Potassium, mmol/L | 3.97 ± 0.96 | 4.01 ± 0.73 | 4.30 ± 1.22 |
| Chloride, mmol/L | 107.75 ± 9.74 | 108.72 ± 9.11 | 109.81 ± 9.13 |
| **Comorbidities** |  |  |  |
| Congestive heart failure, n (%) | 14 (36.84) | 12 (32.43) | 11 (28.95) |
| Atrial fibrillation, n (%) | 8 (21.05) | 7 (18.92) | 6 (15.79) |
| Renal disease, n (%) | 8 (21.05) | 7 (18.92) | 12 (31.58) |
| Hepatic disease, n (%) | 3 (7.89) | 5 (13.51) | 8 (21.05) |
| COPD, n (%) | 2 (5.26) | 4 (10.81) | 2 (5.26) |
| Coronary heart disease, n (%) | 19 (50.00) | 13 (35.14) | 11 (28.95) |
| Stroke, n (%) | 9 (23.68) | 9 (24.32) | 7 (18.42) |
| Cancer, n (%) | 2 (5.26) | 4 (10.81) | 9 (23.68) |
| Respiratory failure, n (%) | 21 (55.26) | 17 (45.95) | 16 (42.11) |
| ARDS, n (%) | 1 (2.63) | 0 (0.00) | 2 (5.26) |
| Pneumonia, n (%) | 30 (78.95) | 32 (86.49) | 25 (65.79) |
| Sepsis, n (%) | 31 (81.58) | 35 (94.59) | 36 (94.74) |
| AKI, n (%) |  |  |  |
| 1 | 16 (42.11) | 14 (37.84) | 18 (47.37) |
| 2 | 2 (5.26) | 6 (16.22) | 3 (7.89) |
| 3 | 20 (52.63) | 17 (45.95) | 17 (44.74) |
| **Scoring systems** |  |  |  |
| SAPSII | 49.39 ± 12.52 | 50.54 ± 15.55 | 52.29 ± 16.22 |
| SOFA | 10.78 ± 3.58 | 9.86 ± 3.92 | 10.29 ± 3.26 |
| Use of vasoactive drugs, n (%) | 24 (63.16) | 23 (62.16) | 26 (68.42) |
| Renal replacement therapy, n (%) | 10 (26.32) | 12 (32.43) | 14 (36.84) |
| Length of stay in ICU, day | 12.71 ± 13.60 | 13.46 ± 14.28 | 16.11 ± 15.76 |
| Hospital mortality, n (%) | 3 (8.11) | 6 (15.79) | 13 (34.21) |
| 30-day mortality, n (%) | 16 (43.24) | 17 (44.74) | 17 (44.74) |
| 90-day mortality, n (%) | 18 (75.00) | 22 (73.33) | 23 (79.31) |

**Abbreviations:** SBP: systolic blood pressure; DBP: diastolic blood pressure; MAP: mean arterial pressure; RA: the ratio of red cell volume distribution width to albumin; RDW: red cell volume distribution width; CRP: C-reactive protein; PT: prothrombin time; INR: international normalized ratio; APTT: activated partial thromboplastin time; BUN: blood urea nitrogen; COPD: chronic obstructive pulmonary disease; ARDS: acute respiratory distress syndrome; AKI: acute kidney injury; SAPSII: simplified acute physiology score II; SOFA: sequential organ failure assessment; ICU: intensive care unit.
